# In Silico-Based Repositioning of Phosphinothricin as a Novel Technetium-99m Imaging Probe with Potential Anti-Cancer Activity

**DOI:** 10.3390/molecules23020496

**Published:** 2018-02-23

**Authors:** Tamer M. Sakr, Mohammed A. Khedr, Hassan M. Rashed, Maged E. Mohamed

**Affiliations:** 1 Radioactive Isotopes and Generator Department, Hot Labs Center, Atomic Energy Authority, Cairo 13759, Egypt; tamer_sakr78@yahoo.com; 2 Pharmaceutical Chemistry Department, Faculty of Pharmacy, October University of Modern Sciences and Arts (MSA), Giza 12111, Egypt; 3 Department of Pharmaceutical Chemistry, Faculty of Pharmacy, Helwan University, Ein Helwan, Cairo 11795, Egypt; 4 College of Clinical Pharmacy, King Faisal University, Al-Hasaa 31982, Kingdom of Saudi Arabia; maged789@hotmail.com; 5 Labeled Compounds Department, Hot Labs Center, Atomic Energy Authority, Cairo 13759, Egypt; H_rashed@yahoo.com; 6 Department of Pharmacognosy, Faculty of Pharmacy, University of Zagazig, Zagazig 44519, Egypt

**Keywords:** in-silico, repositioning, technetium-99m, cancer imaging, phosphinothricin, molecular docking

## Abstract

l-Phosphinothricin (glufosinate or 2-amino-4-((hydroxy(methyl) phosphinyl) butyric acid ammonium salt (AHPB)), which is a structural analog of glutamate, is a recognized herbicide that acts on weeds through inhibition of glutamine synthetase. Due to the structural similarity between phosphinothricin and some bisphosphonates (BPs), this study focuses on investigating the possibility of repurposing phosphinothricin as a bisphosphonate analogue, particularly in two medicine-related activities: image probing and as an anti-cancer drug. As BP is a competitive inhibitor of human farnesyl pyrophosphate synthase (HFPPS), in silico molecular docking and dynamic simulations studies were established to evaluate the binding and stability of phosphinothricin with HFPPS, while the results showed good binding and stability in the active site of the enzyme in relation to alendronate. For the purpose of inspecting bone-tissue accumulation of phosphinothricin, a technetium (^99m^Tc)–phosphinothricin complex was developed and its stability and tissue distribution were scrutinized. The radioactive complex showed rapid, high and sustained uptake into bone tissues. Finally, the cytotoxic activity of phosphinothricin was tested against breast and lung cancer cells, with the results indicating cytotoxic activity in relation to alendronate. All the above results provide support for the use of phosphinothricin as a potential anti-cancer drug and of its technetium complex as an imaging probe.

## 1. Introduction

Bone-seekers are a group of complexes of radioisotopes that tend to accumulate in bones when they are introduced into the body. Bone-seekers have been used for more than 30 years in nuclear medicine to provide convenient and effective means for monitoring disease progression, improving the quality of life for patients with diseases, such as cancer, or for radio-imaging purposes [1]. The mechanisms involved in the uptake of these complexes into bone tissue are still unclear and under investigation. These mechanisms can involve simple chemisorption onto bone minerals. However, bone-seeking radiopharmaceuticals have been developed to exhibit maximum affinity to the inorganic compartments of bone [2]. Technetium-99m is one of the well-known rare earth radioisotopes used in bone-seeker complexes. This compound noticeably reduces patients’ radiation exposure and shows more favorable physical decay characteristics, which leads to increased sensitivity and resolution and improved diagnostic efficacy [3]. 

**Figure 1:**
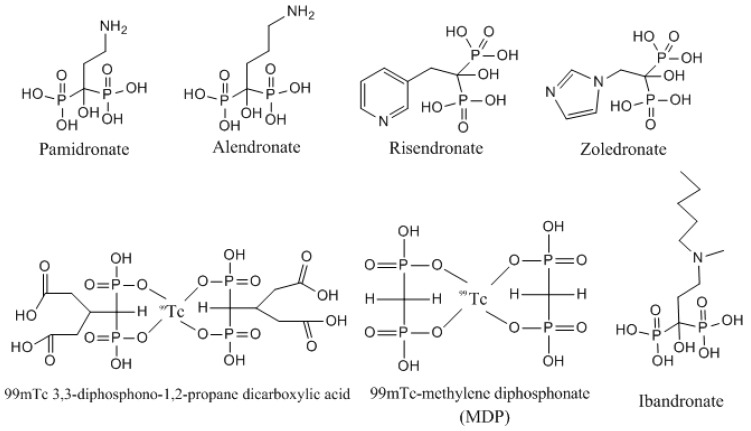

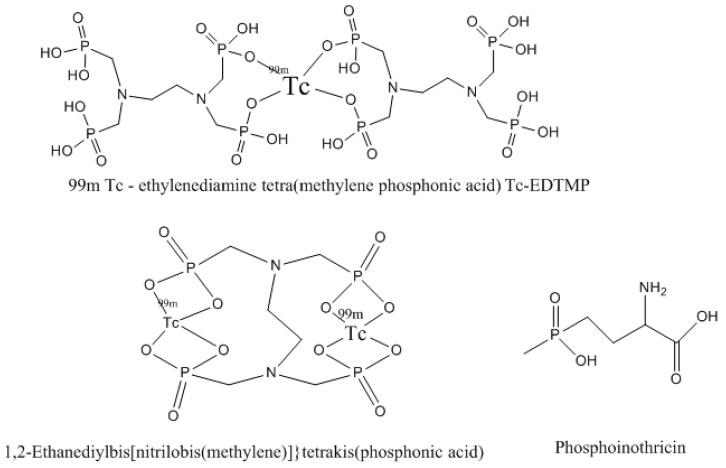
Chemical structures of some of widely-used bisphosphonates and ^99m^Tc-complex radiopharmaceuticals.

In order for bone-seekers to be targeted, they require suitable bone-seeking ligands or tracers. After administration, these ligands carry the radionuclide to the binding sites of bone tissue. Therefore, bone localization is related to properties of the ligand or tracer, while the radionuclide mainly accounts for the molecular imaging of the compound [4]. Bisphosphonates (BPs) are a group of well-known drugs, which are considered the backbone in the treatment of osteoporosis. BPs show high affinity for bone tissues and inhibit bone resorption by being selectively taken up and adsorbed to the mineral surfaces in bone [5]. BPs can mainly be classified into two groups: simpler non-nitrogen containing BPs, such as etidronate and clodronate; and the most potent nitrogen-containing BPs, including pamidronate, alendronate, risedronate, ibandronate and zoledronate (Figure 1) [6]. The high affinity of BPs for bone tissue make them very suitable to be used as tracers or ligands in a bone-seeker complex with radioisotopes, such as ^99m^Tc. The examples of ^99m^Tc-BP complexes are ^99m^Tc-methylene diphosphonate (^99m^Tc-MDP), ^99m^Tc-3,3-diphosphono-1,2-propane dicarboxylic acid (^99m^Tc -DPD), ^99m^Tc-3,3-1,2-ethanediylbis[nitrilobis-(methylene)]tetrakis-phosphonic acid (^99m^Tc-EDTMP) and ^99m^Tc-TEDP (Figure 1) [7,8]. These complexes are involved in routine bone imaging for diagnosis and evaluation of primary tumor uptake and checking for bone metastases [9]. These agents are non-hydrolysable analogues of pyrophosphate. The phosphorus–carbon–phosphorus backbone results in chelation of calcium ions and high affinity for bone mineral. BPs have high selectivity for osteoclasts due to their ability to target bone. The real mechanism of BPs involves the competitive inhibition of human farnesyl pyrophosphate synthase (HFPPS) [10]. The anti-cancer activity of BPs is well recognized and reviewed in many studies [11]. BPs are accepted as treatment for malignant bone disease because they are efficient inhibitors of osteoclast-mediated bone resorption [12]. In women with advanced breast cancer and bone metastases, BPs reduce the incidence of hypercalcemia and skeletal morbidity [13]. The concept of repurposing BPs for the treatment of different types of cancer, including bone and breast cancer, is now a hot spot for scientific investigation in the scientific world [14].

l-Phosphinothricin (l-homoalanine-4-yl-(methyl)-phosphinic acid or glufosinate) is the active ingredient of the non-selective herbicide BASTA. Phosphinothricin, which inhibits glutamine synthetase [15], is a structural analog of glutamate. The result is the rapid accumulation of a high concentrations of ammonia and depletion of glutamine in the plant. This effect leads to a rapid decline of photosynthetic CO_2_-fixation, resulting in plant death. The results of several toxicological studies show that phosphinothricin are safe for users, with an LD_50_ of 2170 mg/kg after oral administration in rats [15,16,17]. The drug is considered theoretically safe for humans, as its target enzyme, glutamine synthetase, is not found in humans or animals. The chemical structure of phosphinothricin exhibits structural similarity with BPs, which have encouraged us to investigate their potential as bone-seekers in nuclear imaging and their anti-cancer activity. 

Therefore, the present study aimed to reposition phosphinothricin using the in silico studies to predict its HFPPS affinity, in vitro evaluation of its anti-cancer activity against breast and lung cancer and in vivo studies, based on radiolabeling approach, to evaluate the ^99m^Tc-phosphinothricin complex as a novel bone scintigraphy radiopharmaceutical. 

## 2. Results and Discussion

### 2.1. Molecular Docking 

The anti-cancer effect of BPs against breast cancer, lung cancer and their metastases has been confirmed in previous studies [10,11]. Many structural activity relationships were conducted on BPs and revealed that the number of carbon atoms in the side chain should not be more than five carbons. The dual effect of these drugs is very interesting, as it includes high affinity to bone and anti-cancer effect. However, BPs have some side effects, such as osteonecrosis of the jaw, severe suppression of bone turnover, low calcium level, change in the kidney functions and digestive problems. Furthermore, they cannot be used for cancer treatment alone and are usually used in combination. For the discovery and/or development of novel anti-cancer agents, which can act by the same selective mechanism without causing any harmful effects, many trials have been conducted for developing novel derivatives of BPs.

**Figure 2:**
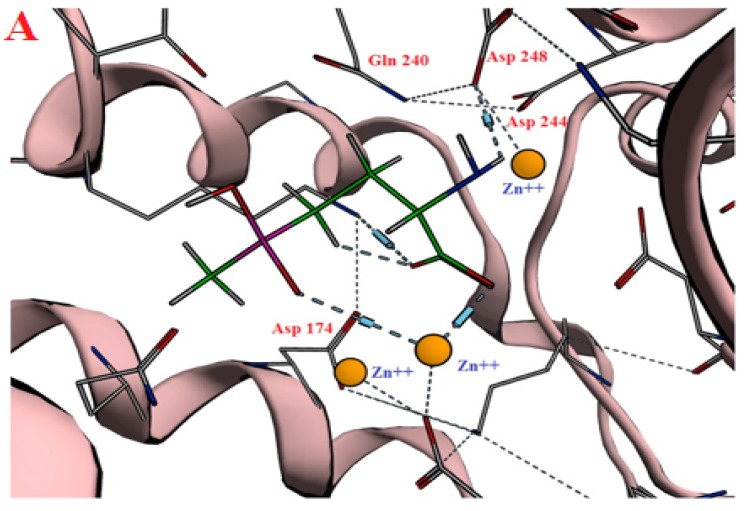

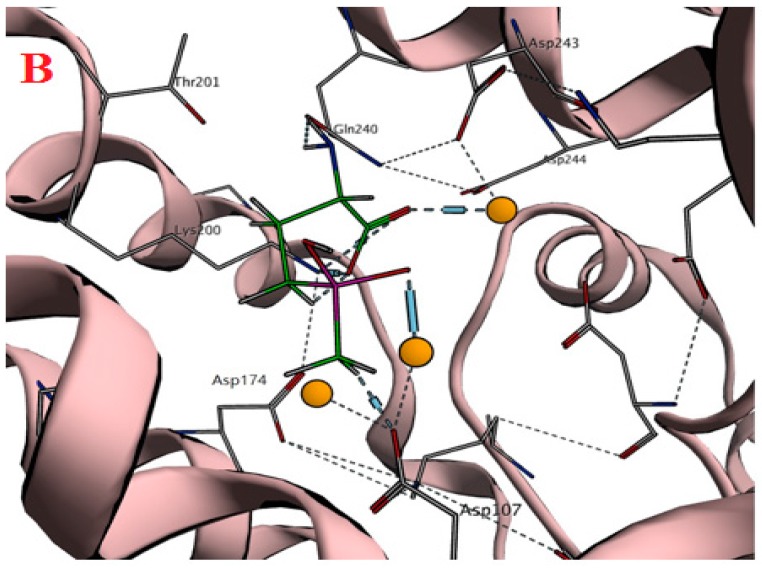
The best binding modes of phosphinothricin inside HFPPS binding site.

**Figure 3:**
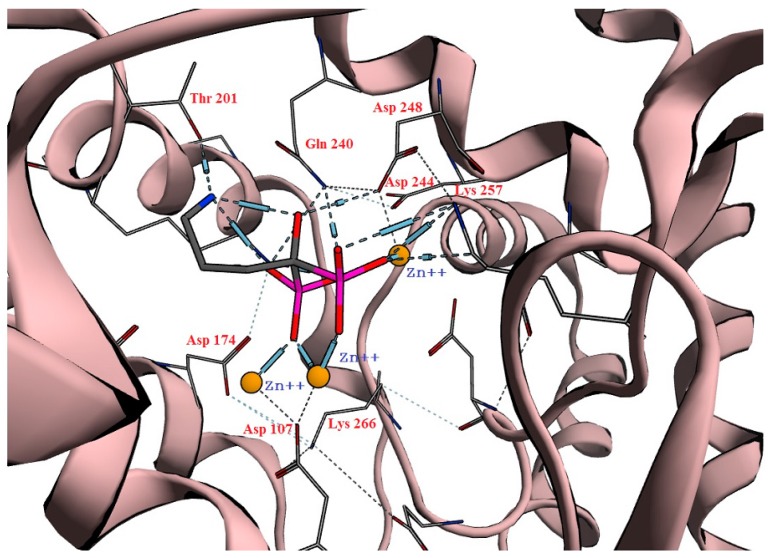
The best orientation pose of alendronate in the active site.

BPs act by inhibition of human farnesyl pyrophosphate synthase (HFPPS) through binding to the catallytic active site and coordinating with the three divalent metal ions at this site. The similarity of structures for different analogues with an isosteric group for the phosphonate was studied. As a result of a long screening process using many compounds with structural similarity to BPs, phosphinothricin was suggested. Phosphinothricin has a carboxylic moiety instead of the phosphonate. In addition, it has an amino group that features in all nitrogenous BP in addition to a methyl phosphonate moiety. To answer our questions, we started with an in silico docking study of phosphinothricin against HFPPS was performed with comparisons made to alendronate, a well-known BP that has a similar structure. The docking study showed that phosphinothricin contained two well-orientated positions, one of which showed three hydrogen bonds with most of the important residues at the active site, such as Asp248, Gln240 and Asp174 in addtion to three coordinates shared with Zn^+2^ (Figure 2A). Another position showed an additional hydrogen bond with Asp107 and another electrostatic bond with Lys200 (Figure 2B). The free energy of binding (ΔG) of phosphinothricin was −25.60 (kcal/mol) with an in silico affinity (pki) of 37.54 when compared with alendronate, which had a ΔG of −27.61 (kcal/mol) with an in silico affinity (pki) of 40.54 (Table 1). The predicted orientation of alendronate included hydrogen bonding with Asp248, Gln240, Thr201, Lys257, Asp107 and Asp174 (Figure 3).

### 2.2. Docking of Tc-Phosphinothricin Complex

The predicted complex structures of both Tc-phosphinothricin and Tc-alendronate were built, minimized and prepared for docking. We found a ΔG of −10.12 kcal/mol and −8.95 kcal/mol for the Tc-phosphinothricin and Tc-alendronate complex, respectively (Table 1). These results demonstrate that Tc-phosphinothricin has a better fit compared to Tc-alendronate, which was also confirmed by computing the in silico affinity (pki) for both complexes, which were 21.74 pki and 22.85 pki for the Tc-AHPB and Tc-alendronate complex, respectively.

**Figure 4:**
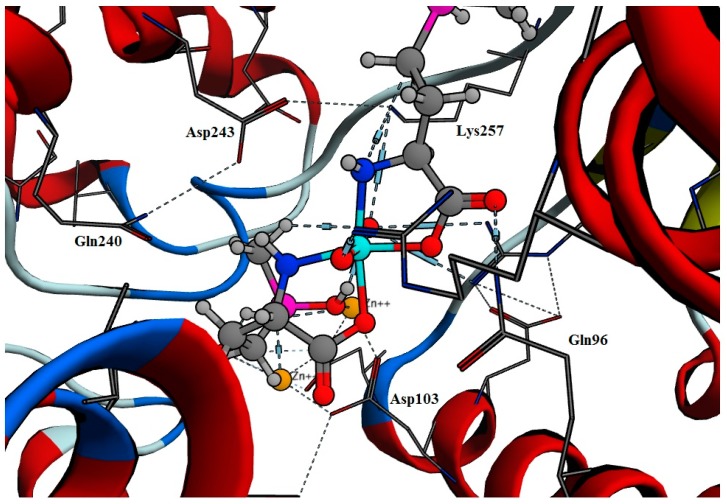
The orientation of Tc- phosphinothricin complex.

**Figure 5:**
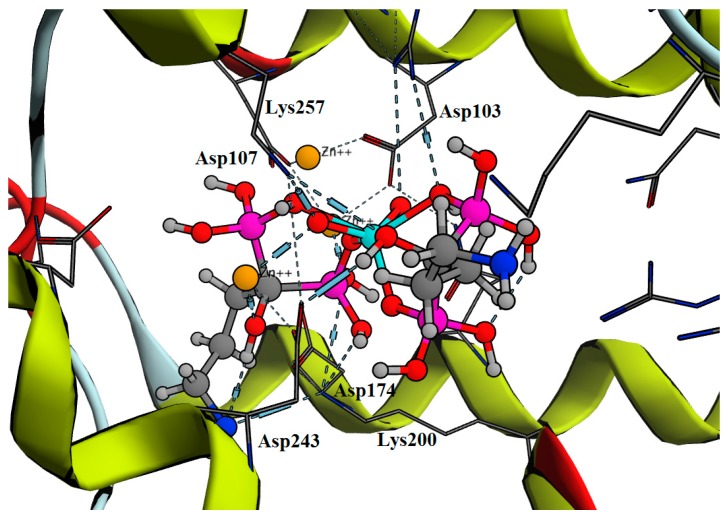
The predicted orientation of Tc-alendronate complex.

The predicted mode of binding for Tc-phosphinothricin included hydrogen bonds with Lys257, Gln96 and Asp103 in addition to coordinating with the three Zn^+2^ ions (Figure 4). On the other hand, Tc-alendronate complex showed hydrogen bonds with Lys257, Gln96, Asp103 and Asp174 (Figure 5).

### 2.3. Molecular Dynamics

**Figure 6:**
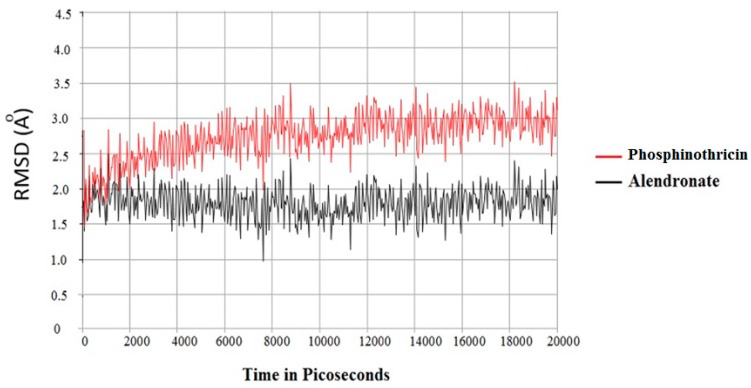
Molecular dynamic simulations of both phosphinothricin and alendronate showing the calculated RMSD (Ǻ).

The best orientated was achieved from the docking of both phosphinothricin and alendronate, which was stored in the active site of HFPPS and saved as a pdb file. MD simulations of these complexes were conducted to evaluate the binding stability of these compounds. The trajectory file was analyzed, which showed that alendronate had lower deviation than phosphinothricin. Alendronate had a RMSD value of 2.0, with further oscillation between 1.5 and 2.0 after a period of time. On the other hand, phosphinothricin oscillations illustrated an increase from a RMSD of 2.0 at the same binding site to reach a value of 2.5, before becoming steady at a RMSD of 3.0 (Figure 6).

### 2.4. Molecular Dynamics Simulations of Tc-Phosphinothricin and Tc-Alendronate Complexes

**Figure 7:**
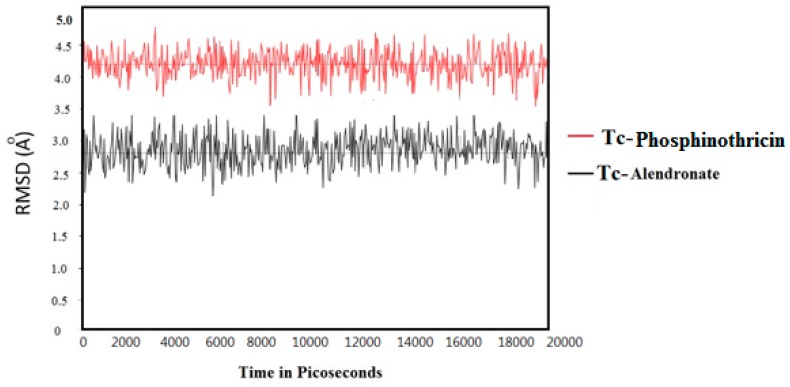
The computed RMSD (Ǻ) of ^99m^TC complex of both phosphinothricin and alendronate.

The MD simulations of Tc-phosphinothricin and Tc-alendronate complexes were also conducted to verify if the Tc-complex would affect the binding for a long period of time. According to the results, Tc-alendronate showed more stability with a low RMSD of 3.0, while Tc-phosphinothricin showed stability at a RMSD of 4.3 (Figure 7).

### 2.5. Radiolabeling of Phosphinothricin.

**Figure 8:**
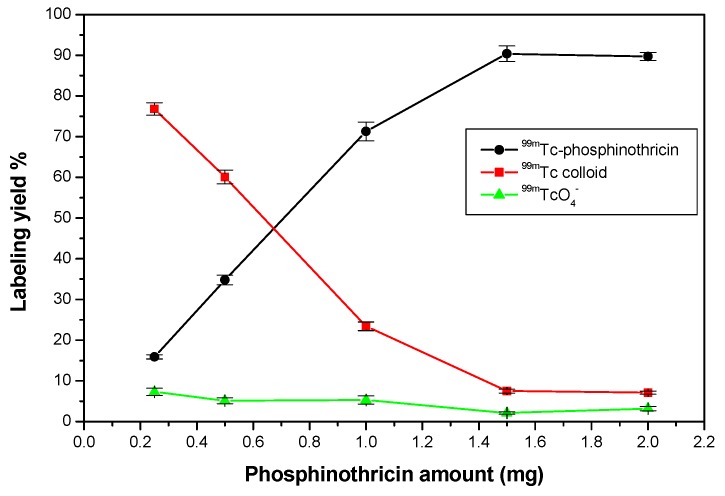
Radiochemical yield of ^99m^Tc-phosphinothricin as a function of phosphinothricin amount. Reaction conditions: 10 µg of SnCl_2_·2H_2_O, 100 µL (7.2 MBq) of ^99m^TcO_4_^−^ solution, at a pH 8 for 15 min.

**Figure 9:**
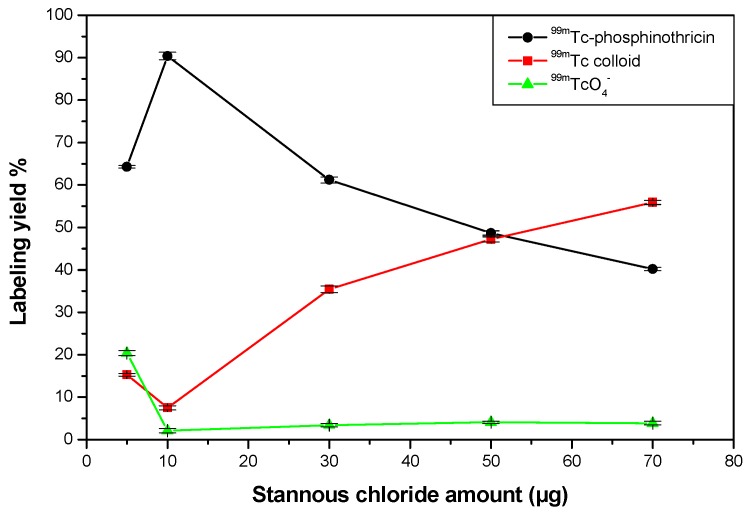
Radiochemical yield of ^99m^Tc-Phosphinothricin as a function of SnCl_2_·2H_2_O amount. Reaction conditions: 1.5 mg of phosphinothricin, 100 µL (7.2 MBq) of ^99m^TcO_4_^−^ solution, at a pH 8 for 15 min.

**Figure 10:**
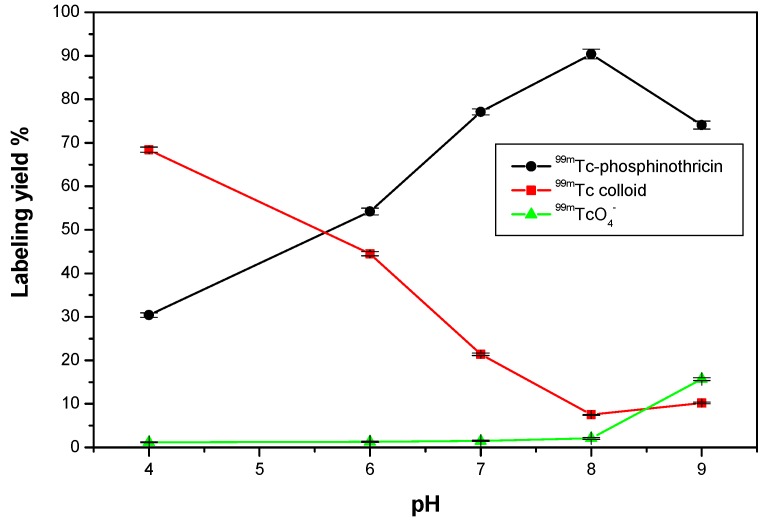
Radiochemical yield of ^99m^Tc-phosphinothricin as a function of reaction pH. Reaction conditions: 1.5 mg of phosphinothricin, 10 µg SnCl_2_·2H_2_O, and 100 µL (7.2 MBq) of ^99m^TcO_4_^−^ solution, for 15 min.

**Figure 11:**
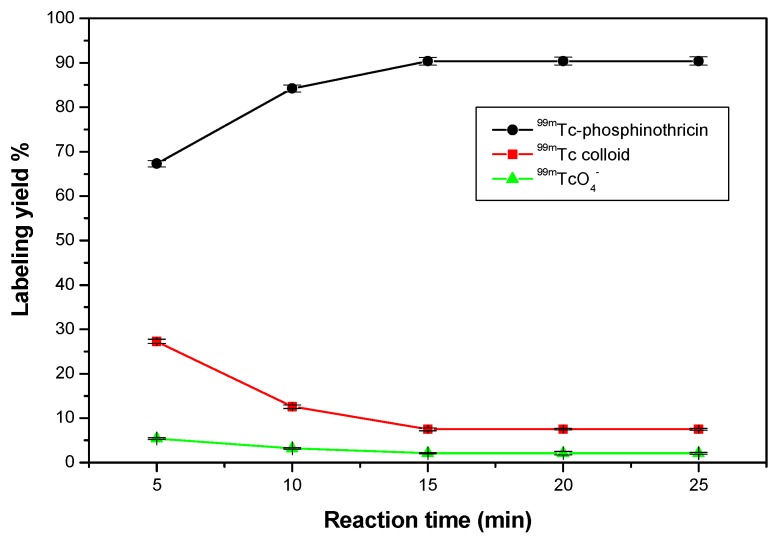
Radiochemical yield of ^99m^Tc-phosphinothricin as a function of reaction time. Reaction conditions: 1.5 mg of phosphinothricin, 10 µg SnCl_2_·2H_2_O, and 100 µL (7.2 MBq) of ^99m^TcO_4_^−^ solution, at pH of 8.

An optimum radiochemical yield of 90.5% ± 0.51% was achieved using 1.5 mg of phosphinothricin and 10 µg of SnCl_2_·2H_2_O. Radiolabeling was performed at room temperature (27 ± 3 °C) for 15 min at a pH of 8 (Figure 8, Figure 9, Figure 10 and Figure 11). 

#### 2.5.1. Effect of Phosphinothricin Content

When a small amount of phosphinothricin (0.25 mg) was used, it was insufficient to form complexes with all the reduced technetium present, which was subsequently converted to reduced hydrolyzed ^99m^Tc colloid. This resulted in a low radiochemical yield of 15.9% [18]. The maximum labeling yield of 90.5% was obtained by increasing the phosphinothricin amount to 1.5 mg. Further increases in the amount of phosphinothricin above 1.5 mg did not have any major impact on the labeling yield (Figure 8).

#### 2.5.2. Effect of SnCl_2_·2H_2_O Content

Incomplete reduction of free ^99m^TcO_4_^−^ occurred with small amounts of SnCl_2_·2H_2_O used, resulting in a percentage of low ^99m^Tc-phosphinothricin (64.3% at 5 µg). By increasing the amount of SnCl_2_·2H_2_O to 10 µg, the labeling yield was increased to 90.4% (Figure 9).

When too much SnCl_2_·2H_2_O was added, the labeling yield decreased (40.2% at 70 µg) and the amount of colloid increased. This is because most of phosphinothricin became complexed with ^99m^Tc, so the free ^99m^Tc was reduced to insoluble technetium (IV) TcO_2_·xH_2_O in the absence of phosphinothricin [19]. Furthermore, the stannous chloride excess forms stannous hydroxide colloid Sn(OH)_3_^−^ in the basic medium [20,21].

#### 2.5.3. Effect of pH of the Reaction Medium

The labeling yield of ^99m^Tc-phosphinothricin increased with increasing pH of the reaction medium (Figure 10). At a pH of 4, the labeling yield was low (30.4%) due to protonation of lone pair carrying oxygen atoms of phosphinothricin, which compete with technetium for binding to the lone pair [22]. The maximum labeling yield (90.4%) was obtained at a pH of 8, at which point phosphinothricin combined with all the reduced technetium [23]. Further increases in pH decreased the labeling yield to 74.1% at a pH of 9. This is because SnCl_2_·2H_2_O readily precipitates at high pH values, while this high pH also favors the formation of free ^99m^TcO_4_^−^ and insoluble reduced hydrolyzed ^99m^Tc colloid in an alkaline medium [24,25].

#### 2.5.4. Effect of Reaction Time

A short reaction time (5 min) was insufficient to form the ^99m^Tc-phosphinothricin complex, which lead to a low labeling yield (67.3%). Increasing the reaction time to 15 min caused an increase in the radiochemical yield to 90.4%, which remained constant with a further increase in the reaction time to 25 min (Figure 11).

#### 2.5.5. In Vitro Stability of ^99m^Tc-phosphinothricin Complex

**Figure 12:**
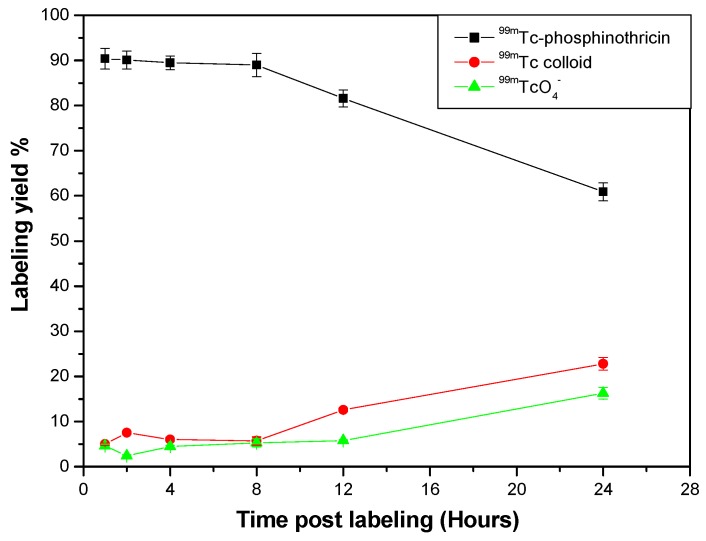
In vitro stability of ^99m^Tc-phosphinothricin complex.

Hydrolysis, oxidation or γ-radiolysis of ^99m^Tc-radiopharmaceuticals may occur during storage. Therefore, in vitro stability of such radiopharmaceutical compounds determines the most suitable time during which the preparation could be used [18]. The ^99m^Tc-phosphinothricin complex showed in vitro stability for more than 8 h after labeling, with a radiochemical yield of 90.4% until 8 h had passed (Figure 12).

#### 2.5.6. Biological Distribution Study

The biodistribution of ^99m^Tc-phosphinothricin complexes in different body organs and fluids of mice is shown in Table 2. Bones showed rapid, high and sustained uptake of the radioactivity starting 10–90 min (36.13 ± 1.5 and 52.62 ± 2.6% ID, respectively) post-injection, which shows the high affinity of ^99m^Tc-phosphinothricin complex for bones. Such rapid efficient bone uptake would lessen the burden on patients in terms of the total length of the examination and the dose of radiation absorbed. Besides, the long retention of the ^99m^Tc-phosphinothricin complexes in bones indicates its high biological stability. The blood radioactivity decreased rapidly (from 41.51 ± 2.5 at 10 min to 5.16 ± 1.2 at 25 min) as a result of bone uptake and clearance through the kidneys 25 min post injection. The radioactivity levels of liver and intestine at different time intervals indicate that ^99m^Tc-phosphinothricin excretion occurs through both renal and hepatobiliary systems.

### 2.6. In Vitro Evaluation of the Anti-Cancer Activity

The in vitro cytotoxity of phosphinothricin against the breast cancer cell line MCF-7 showed an IC_50_ of 32.6 μg/mL, much better than that of alendronate with an IC_50_ of 89.4 μg/mL. The anti-cancer effect on lung cancer cell line A-549 was reversed, where phosphinothricin had an IC_50_ of 45.8 μg/mL and alendronate showed an IC_50_ of 19 μg/mL.

**Figure 13:**
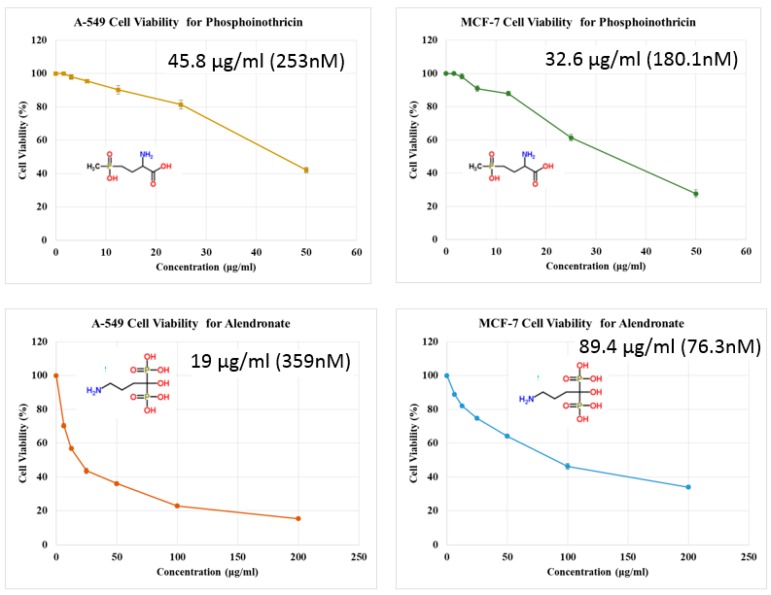
In vitro anti-cancer evaluation of phosphinothricin against breast cancer MCF-7 cell line (B&D) and lung cancer A-549 cell line (A&C).

As a result, we can conclude that phosphinothricin revealed promising in vitro anti-cancer activity against both breast and lung cancer cell lines (Figure 13).

### 2.7. Bone Seeking, Imaging Probe and Anti-Cancer Activities

Due to the structural similarity between phosphinothricin and BPs, especially alendronate, phosphinothricin was proposed to exert similar pharmaceutical and medicinal properties to alendronate, particularly its bone-seeking, image-probing and anti-cancer properties. In order to test this hypothesis, phosphinothricin was introduced as two pharmaceutical preparations: raw drug and Tc-phosphinothricin complex. The anti-cancer properties of the raw drug were investigated and phosphinothricin showed good cytotoxic activity relative to its structural analogue, alendronate. Tc-phosphinothricin complex was produced in a stable form, which allowed for the examination of the bone-seeking activity of phosphinothricin as well as the possibility of using this complex as an imaging probe. The results indicated a high absorption pattern of Tc-phosphinothricin complex into bone tissue, recommending its use as a probe. The high concentration of phosphinothricin in bone tissue and its verified cytotoxic activity suggest its potential use in bone cancers. Phosphinothricin is cheap, easily sourced, non-toxic and very well studied due to its long history of being used as an herbicide. This study demonstrated the ability of phosphinothricin to be used for the treatment of cancer in breast, lung and maybe bone cancer. At the same time, we produced a new pharmaceutical preparation, Tc-AHPB complex, which could be used as an image probe. 

## 3. Material and Methods

### 3.1. Materials

Chemicals were analytical grade and purchased from Sigma-Aldrich (Cairo, Egypt). Male Swiss Albino mice (20–25 g) were purchased from the National Cancer Institute (Doki, Egypt). Technetium-99m was eluted as ^99m^TcO_4_^−^ from ^99m^Mo/^99m^Tc generator (activity: 1Ci, Radioisotope Production Facility, Cairo, Egypt). Phosphinothricin (glufosinate-ammonium, or 2-amino-4-(hydroxymethyl-phosphinyl butyric acid ammonium salt or l-homoalanine-4-yl-(methyl)-phosphinic acid or AHPB), CAS Number 77182-82-2, Molecular Weight 198.16, MP 215 °C) was purchased from Sigma-Aldrich (product number 45520). Molecular Operating Environment MOE 2016.08 was purchased from Chemical Computing Group Inc. (Montreal, QC, Canada) [26].

### 3.2. Molecular Docking of the Phosphinothricin

The crystal structure of HFPPS in complex with alendronate was downloaded from the Protein Data Bank (pdb code = 2f92) [27]. It was obtained by the X-ray diffraction method with resolution of 2.15 Å and R-value free of 0.289. All coordinates were derived from pdb and all interactions were observed with the conserved residues; Thr201, Gln240, Asp248, Asp244, Lys257, Asp174, Asp107 and Lys266. The docking protocol used the triangle method as a placement method with timeout of 300 s, and number of return poses as 1000. London dG was used as a rescoring method. Force field was used as a refinement method by applying MMFF94x

### 3.3. Molecular Dynamics Simulations 

All molecular dynamic simulations were conducted using MOE 2016.08. The best conformations from each docking process of both alendronate and phosphinothricin were kept inside the active site. The quality of the temperature-related factors, protein geometries, and electron density was tested. All hydrogens were added and energy minimization was computed. The solvent molecules that were in the system were deleted before solvation; salt atoms were added to ensure complete neutralization of the biomolecular system. The solvent atoms were added to surround the biomolecular system (protein-ligand complex) in a spherical shape. Amber 10:EHT was selected as a force field in the potential setup step. All Van der Waals forces, electrostatics, and restraints were enabled. The heat was adjusted in order to increase the temperature of the system from 0 to 300 K that was followed by equilibration and production for 300 ps; cooling was then initiated until to 0 K was reached. The simulation was conducted over 20 ns period of time (20,000 ps).

### 3.4. Preparation of ^99m^Tc-Phosphinothricin (^99m^Tc-AHPB) Complex

#### 3.4.1. Labeling Procedure

Direct labeling technique was used to radiolabel phosphinothricin by ^99m^Tc under reductive conditions using SnCl_2_·2H_2_O as a reducing agent [28]. Different labeling reactions were done in evacuated 10 mL penicillin vials to determine the optimum labeling conditions. Different amounts of phosphinothricin (0.25–2 mg) dissolved in 1 mL of N_2_-purged distilled water were added followed by adding (5–70 µg) SnCl_2_·2H_2_O. Then, 100 µL of ^99^Mo/^99m^Tc generator eluate containing 7.2 MBq of ^99m^TcO_4_^−^ was added to each vial. Different reaction medium pH values (4–9) were studied using different amounts of 0.1 N NaOH or 0.1 N HCl solutions. Effect of reaction time on labeling process was studied in range of 5–25 min. 

#### 3.4.2. Radiochemical Yield of ^99m^Tc-Phosphinothricin Complex

The effect of different labeling conditions on radiochemical yield was judged by paper chromatography to determine the percentage of ^99m^Tc-phosphinothricin complex. Ascending paper chromatography (using strips of No. 1 Whatman paper) was performed. Dual solvent systems were used as mobile phases to determine the labeling yield. Acetone was used to determine the free ^99m^TcO_4_^−^ percentage where its R_f_ is 0.95 while ^99m^Tc-phosphinothricin and ^99m^Tc-colloidal remain in the origin, while ethanol:water:ammonium hydroxide mixture (2:5:1, *v*/*v*/*v*) was used to determine the ^99m^Tc-colloidal impurities where its R_f_ is 0 while ^99m^Tc-phosphinothricin and ^99m^TcO_4_^−^ move to the front [29,30,31].

#### 3.4.3. In Vitro Stability of ^99m^Tc-Phosphinothricin Complex

^99m^Tc-phosphinothricin complex was evaluated up to 24 h, after labeling for its in vitro stability. At different time intervals up to 24 h, aliquots from the reaction mixture were assessed using paper chromatography to determine the radiochemical yields.

#### 3.4.4. Biodistribution Study

All the biodistribution studies were performed according to the animal ethics guidelines of the Egyptian Atomic Energy Authority (ethical approval EAEA/23/2017). The bone imaging ability of ^99m^Tc-phosphinothricin complex was studied in male Swiss Albino mice. ^99m^Tc-phosphinothricin complex (0.1 mL) containing 185–1850 kBq was I.V. injected in the mice tail veins after filtration through a 0.22 μm Millipore filter for sterilization and removing the colloidal impurities [32,33]. The animals were anesthetized by chloroform at the predesigned time intervals (10, 25, 60, 90 and 120 min post injection). Since it’s impossible to collect all blood, bone and muscles of mice, samples of each of them was collected and the total radioactivity level for each is calculated depending on their percentage to the total mice body weight (7, 10 and 40%, respectively) [34,35,36,37]. Other body organs were separated, weighed and their radioactivity levels were assayed using a NaI(Tl) γ-ray scintillation counter. % Injected dose per organ (% ID ± S.D.) in three mice for each time point were estimated. 

#### 3.4.5. In Vitro Evaluation of the Anticancer Activity Using a Viability Assay 

For cytotoxicity assays against breast cancer MCF-cell line and lung cancer A-549 cell line the cells were obtained from the VACSERA culture unit (Cairo, Egypt). The cells were seeded in 96-well plates at a cell concentration of 1 × 10^4^ cells per well in 100 µL of growth medium. Fresh medium containing different concentrations of the test sample was added 24 h after seeding. Serial two-fold dilutions of the tested chemical compound were added to confluent cell monolayers dispensed into 96-well, flat-bottomed microliter plates (Falcon, NJ, USA) using a multichannel pipette. The microliter plates were incubated at 37 °C in a humidified incubator with 5% CO_2_ for a period of 48 h. Three wells were used for each concentration of the test sample. Control cells were incubated without test sample and with or without DMSO. The little percentage of DMSO present in the wells (maximal 0.1%) was found not to affect the experiment. After incubation of the cells at 37 °C, various concentrations of sample were added, and the incubation was continued for 24 h and viable cells yield was determined by a colorimetric method. In brief, after the end of the incubation period, media were aspirated and the crystal violet solution (1%) was added to each well for at least 30 min. The stain was removed and the plates were rinsed using tap water until all excess stain is removed. Glacial acetic acid (30%) was then added to all wells and mixed thoroughly, and then the absorbance of the plates were measured after gently shaken on microplate reader (TECAN, Inc., Morrisville, NC, USA), using a wavelength of 490 nm. All results were corrected for background absorbance detected in wells without added stain. Treated samples were compared with the cell control in the absence of the tested compounds. All experiments were carried out in triplicate. The cell cytotoxic effect of each tested concentration was calculated. The optical density was measured with the microplate reader (SunRise, TECAN, Inc., Morrisville, NC, USA) to determine the number of viable cells and the percentage of viability was calculated as [1 − (ODt/ODc)] × 100% where ODt is the mean optical density of wells treated with the tested sample and ODc is the mean optical density of untreated cells. The relation between surviving cells and drug concentration is plotted to get the survival curve of each tumor cell line after treatment. The 50% inhibitory concentration (IC_50_), the concentration required to cause toxic effects in 50% of intact cells, was estimated from graphic plots of the dose response curve for each conc. using Graphpad Prism software (San Diego, CA, USA). The biological evaluation was done at the Regional Center for Mycology & Biotechnology, Al-Azhar University.

## 4. Conclusions

Phosphinothricin was repurposed as an anti-cancer drug because of its structural similarity to alendronate. The docking results revealed that phosphinothricin possessed a ΔG of −25.65 kcal/mol with a pki of 37.54, while its Tc-complex showed a ΔG of −10.12 kcal/mol with a pki of 21.74, which confirmed its high affinity when compared to alendronate. The radiolabeling of phosphinothricin showed that bones displayed high and sustained uptake of the radioactivity starting 10–90 min after administration (36.13 ± 1.5 and 52.62 ± 2.6% ID, respectively), which is a another confirmation of its affinity to bone cells. The in vitro anti-cancer screening of phosphinothricin had an IC_50_ of 32.6 μg/mL (180.1 nM) and IC_50_ of 45.8 μg/mL (253 nM) against breast cancer MCF-7 and lung cancer cell line A-549, respectively. The study confirmed the repurposing of phosphinothricin as a novel technetium-99m imaging probe with potential anti-cancer activity.

## Figures and Tables

**Table 1:** Molecular docking results using MOE.

Compound	ΔG (kcal/mol)	Affinity pki	ΔG (Rescoring) (kcal/mol)
Phosphinothricin	−25.65	37.54	−24.75
Alendronate	−27.61	40.54	−26.55
Tc-phosphinothricin complex	−10.12	21.74	−9.10
Tc-Alendronate complex	−8.95	22.85	−7.65

**Table 2:** Biological distribution of ^99m^Tc-phosphinothricin complex in mice as a function of time (%ID/organ ± S.D., n = 3).

Organ/Fluid	Time				
	10 min	25 min	1 h	1.5 h	2 h
Blood	41.51 ± 2.5	5.16 ± 1.2	4.76 ± 0.94	4.69 ± 1.09	4.68 ± 1.1
Kidneys	0.81 ± 0.2	10.04 ± 1.9	5.39 ± 1.05	4.30 ± 0.5	7.20 ± 0.8
Liver	1.44 ± 0.6	3.10 ± 0.9	13.55 ± 1.3	13.33 ± 0.93	15.23 ± 1.3
Spleen	0.32 ± 0.02	0.18 ± 0.2	1.49 ± 0.24	1.00 ± 0.02	0.19 ± 0.07
Intestine	2.21 ± 0.31	6.99 ± 0.46	6.26 ± 0.64	14.54 ± 2.01	34.12 ± 1.88
Stomach	4.80 ± 0.15	2.31 ± 0.52	1.33 ± 0.06	2.07 ± 0.5	4.54 ± 0.7
Lungs	2.37 ± 0.09	5.49 ± 0.33	5.45 ± 0.7	1.86 ± 0.1	2.23 ± 0.21
Heart	0.34 ± 0.07	3.24 ± 0.41	0.77 ± 0.03	1.73 ± 0.06	0.54 ± 0.08
Muscle	10.07 ± 1.1	9.93 ± 2.3	9.01 ± 0.29	3.83 ± 0.86	8.54 ± 1.1
Bone	36.13 ± 1.5	53.56 ± 3.1	51.99 ± 2.4	52.62 ± 2.6	22.73 ± 1.7
